# Four-octyl itaconate activates Keap1-Nrf2 signaling to protect neuronal cells from hydrogen peroxide

**DOI:** 10.1186/s12964-018-0294-2

**Published:** 2018-11-15

**Authors:** Hua Liu, Yu Feng, Min Xu, Jian Yang, Zhichun Wang, Guangfu Di

**Affiliations:** 1 Department of Neurosurgery, Yijishan Hospital, Wannan Medical College, Wuhu, China; 2 Department of Neurosurgery, The First People’s Hospital of Kunshan, Jiangsu University, Suzhou, China; 3 Department of Endocrinology, The Second Affiliated Hospital of Soochow University, Suzhou, China; 4 Department of Neurosurgery, Kunshan Hospital of Traditional Chinese Medicine, Kunshan Affiliated Hospital, Suzhou, China

**Keywords:** Four-octyl itaconate, Keap1-Nrf2 signaling, Neuronal cells, Oxidative stress

## Abstract

**Background:**

Four-octyl itaconate (OI), the itaconate’s cell-permeable derivative, can activate Nrf2 signaling via alkylation of Keap1 at its cysteine residues. The current study tested the potential neuroprotective function of OI in hydrogen peroxide (H_2_O_2_)-treated neuronal cells.

**Methods:**

SH-SY5Y neuronal cells and epigenetically de-repressed (by TSA treatment) primary murine neurons were treated with OI and/or H_2_O_2_. Nrf2 pathway genes were examined by Western blotting assay and real-time quantitative PCR analysis. Neuronal cell death was tested by the LDH and trypan blue staining assays. Apoptosis was tested by TUNEL and Annexin V assays.

**Results:**

In SH-SY5Y neuronal cells and primary murine neurons, OI activated Nrf2 signaling, causing Keap1-Nrf2 disassociation, Nrf2 protein stabilization and nuclear translocation, as well as expression of Nrf2-regulated genes (*HO1*, *NQO1* and *GCLC*) and *ninjurin2* (*Ninj2*). Functional studies showed that OI attenuated H_2_O_2_-induced reactive oxygen species (ROS) production, lipid peroxidation and DNA damage as well as neuronal cell death and apoptosis. shRNA-mediated knockdown, or CRISPR/Cas9-induced knockout of Nrf2 almost abolished OI-induced neuroprotection against H_2_O_2_. Keap1 is the primary target of OI. Keap1 knockout by CRISPR/Cas9 method mimicked and abolished OI-induced actions in SH-SY5Y cells. Introduction of a Cys151S mutant Keap1 in SH-SY5Y cells reversed OI-induced Nrf2 activation and anti-H_2_O_2_ neuroprotection.

**Conclusions:**

OI activates Keap1-Nrf2 signaling to protect SH-SY5Y cells and epigenetically de-repressed primary neurons from H_2_O_2_ in vitro.

**Electronic supplementary material:**

The online version of this article (10.1186/s12964-018-0294-2) contains supplementary material, which is available to authorized users.

## Background

Excessive oxidative stress shall induce neuronal cell injury, which is implicated in the physiological process of aging and a variety of neurodegenerative disease, including Parkinson’s, Huntington’s and Alzheimer’s diseases [1–4]. Central nerve system (CNS) and neuronal cells are especially vulnerable to oxidative stress, possibly due to their high oxygen consumption rate and enrichment in polyunsaturated fatty acids [1–4]. Reactive oxygen species (ROS) accumulation will damage proteins, DNA, and lipid membranes, thereby disrupting neuronal cell functions, causing neuronal cell death and apoptosis [1–4]. Hydrogen peroxide (H_2_O_2_) is produced during the redox process [5–7]. H_2_O_2_ over-production shall induce lipid peroxidation, DNA damage, and neuronal cell death [5–7]. H_2_O_2_ is added to culture neuronal cells in vitro to mimic oxidative injury [5–7].

Nuclear factor E2-related factor 2 (Nrf2) is a key transcription factor regulating expression of a number of antioxidant enzymes, offering significant cellular protection against oxidative stress [8–10]. Once activated, Nrf2 translocates to cell nuclei, and binds to antioxidant response elements (ARE) in the promoter of multiple anti-oxidant and detoxifying enzymes, including *heme oxygenase 1* (*HO-1*), *NAD(P) H quinone oxidoreductase-1* (*NQO1*) and *γ-glutamyl cysteine ligase catalytic subunit* (*GCL*C), promoting their expression [8–10].

In the unstimulated condition, Keap1 (Kelch like ECH-associated protein 1) association with Nrf2 leads to Nrf2 cytoplasmic sequestration and ubiquitin-mediated proteasomal degradation by Cul3 ubiquitin ligase [8–10]. Activated Nrf2 disassociates with Keap1, leading to its stabilization, nuclear translocation and activation [8–10]. Nrf2 activation is shown to protect neuronal cells against oxidative injury [11–13]. Further, upregulation of Nrf2-driven antioxidant enzymes is beneficial in models of neurodegenerative diseases [11–13].

Very recent studies confirmed itaconate as a novel and potent Nrf2 activator [14, 15]. Itaconate directly alkylates Keap1 at its cysteine residues, causing Nrf2-Keap1 disassociation and Nrf2 activation [14]. The cell-permeable itaconate derivative, 4-octyl itaconate (OI), is shown to efficiently promote Nrf2 activation [14]. The potential effect of OI in H_2_O_2_-treated neuronal cells is tested in the present study. Our results show that OI protects neuronal cells from H_2_O_2_ via activation of Nrf2 signaling.

## Material and methods

### Reagents, chemicals and antibodies

Following the previously-described protocol [14] four-octyl itaconate (OI) was synthesized by Ruilu Chemicals (Shanghai, China). H_2_O_2_, anti-(β-)tubulin antibody, puromycin, polybrene, Trypan blue and JC-1 dyes were provided by Sigma-Aldrich (St. Louis, MO). All antibodies were purchased from Abcam (Cambridge, UK) and Cell Signaling Tech (Shanghai, China). The carboxi-2′,7′-dichlorodihydrofluorescein diacetate (carboxy-H2DCFDA) fluorescence dye was purchased from Molecular Probes (Carlsbad, CA). TdT-mediated dUTP nick end labeling (TUNEL) apoptosis detection kit (FITC-labeled) was obtained from Keygen Biotech (Nanjing, China). TRIzol reagents were provided by Biyuntian (Wuxi, China). Cell Counting Kit-8 (CCK-8) was provided by Dojindo Laboratories (Kumamoto, Japan). Cell culture reagents were purchased from Hyclone Co. (Logan, UT). Lipofectamine 2000 was provided by Invitrogen (Shanghai, China).

### Cell culture

Human neuronal SH-SY5Y cells (from Dr. Gao [16]) were cultured in DMEM plus 10% fetal bovine serum (FBS). Before H_2_O_2_ treatment, SH-SY5Y cells were cultured for 5 days with 10 μM retinoic acid (RA) in DMEM plus 10% FBS, 2 mM glutamine, and necessary antibiotics, followed by another 5 days culture in serum-free DMEM with BDNF (brain-derived neurotrophic factor, 50 ng/mL) and glutamine (2 mM) and antibiotics (P/S). As described [16], the primary murine neurons were prepared from E13–E15 embryos of C57 mouse. Neurons were dissociated, counted, and plated in poly-lysine-coated 48-well plates at a density of 1 × 10^5^ cells/well in neurobasal medium plus 2% B27, 500 μM L-glutamine, 20 ng/mL trichostatin A (TSA) and antibiotics (P/S). At day-10 (DIV), over 98% of cells were neurons. The protocol of the study was approved by the Ethics Committee of all authors institutions.

### Cell viability assay

Cells were seeded onto the 96-well plates (2.5 × 10^4^ cells/cm^2^). Following the indicated treatment, cell viability was tested by the CCK-8 assay kit via the recommended procedure. The CCK-8’s optical density (OD) value at 550 nM was recorded.

### Trypan blue staining assay

Following treatment of cells, trypan blue was added to stain the “dead” cells. Cell death percentage was calculated via an automated cell counter (Merck Millipore, Shanghai, China).

### LDH assay

The death of neurons was examined by calculating lactate dehydrogenase (LDH) release to the medium, using a simple two-step LDH enzymatic reaction kit (Takara, Tokyo, Japan). LDH content in the medium was normalized to total LDH (medium LDH plus cellular LDH).

### ROS assay

As previously described [17], cellular ROS content was tested by using the carboxy-H2DCFDA dye. Following treatment, cells were stained with 10 μM of carboxy-H2-DCFDA for 20 min. DCF fluorescence was measured under 485 nm excitation and 525 nm emission using the Fluoroskan Ascent FL machine (Thermo Scientific, Shanghai, China).

### Superoxide assay

The cellular superoxide level was examined using a superoxide assay kit (Beyotime, Wuxi, China) according to the manufacturer’s instructions. Briefly, neuronal cells were cultured onto six-well plates at a density of 3 × 10^5^ cells/well. Following the indicated treatment, the superoxide detection reagent (100 μL/well) was added for 20 min at room temperature. The absorbance, reflecting the superoxide assay, was recorded at 450 nm.

### Lipid peroxidation assay

Following treatment, thiobarbituric acid reactive substances (TBAR) activity was tested to quantify cellular lipid peroxidation level, using the previously-described protocol [17, 18].

### DNA damage assay

The detailed protocol of analyzing DNA damage level was described previously [17, 18]. Briefly, after the indicated treatment, cells were washed and tested by FACS assay to quantify p-γ-H2AX percentage, which reflects DNA damage intensity [19]. The p-γ-H2AX percentage was recorded.

### Real-time quantitative PCR analysis

As previously described [17], Total RNA was extracted by the Trizol reagents. For each condition, five hundred ng of DNA-free total RNA was utilized to perform the reverse transcription with the 2-step RT-PCR kit (Takara Bio, Japan) [20]. Quantitative real-time PCR (qPCR) was performed using the 7500HT Fast Real-Time PCR system (Applied Biosystems). The data presented were normalized to *GAPDH transcripts*. mRNA primers of *human GCLC*, *Nrf2*, *HO1*, *NQO1* and *GAPDH* were described previously [21]. mRNA primers of *murine GCLC*, *Nrf2*, *HO1*, *NQO1* and *GAPDH* were described in the other study [22]. mRNA primers for *Ninj2*, forward, 5’-ATGCGGCTGAAGGCGGTGCTG-3′ and reverse, 5’-TGGCTGCGTTGTTGAGCTGGTTG-3′, were synthesized by Genechem (Shanghai, China).

### Western blotting assay

After treatment, cells/neurons were lysed in SDS lysis buffer [16]. The detailed protocol of Western blotting assay was described previously [23]. Each lane in the SDS-PAGE gene was loaded with exact same amount of quantified protein lysates (30 μg per sample). Same set of lysate samples were run in sister gels to test different proteins when necessary. Nuclear proteins were extracted via the nuclear extraction kit (Sigma, Shanghai, China) by high-speed centrifugation. For data quantification, each band was quantified via the ImageJ software (NIH).

### Immunoprecipitation assay

A total of 600 μg protein lysates per sample were pre-cleared with IgA/G beads (Sigma, Shanghai, China). Endogenous Keap1 was precipitated with anti-Keap1 antibody and protein IgA/G beads (IP). The Keap1-Nrf2 immuno-complex was subjected to Western blotting analysis.

### Caspase-3 activity assay

As previously described [16], 20 μg of cytosolic proteins were incubated with the caspase-3 assay buffer [16] along with the 7-amido-4-(trifluoromethyl) coumarin (AFC)-conjugated caspase-3 substrate (Calbiochem-EMD Millipore, Shanghai, China). After two hours incubation, the release AFC was tested by the Fluoroskan Ascent FL machine under 355 nm excitation and 525 nm emission.

### ssDNA ELISA assay of apoptosis

Following treatment, the cellular content of single strand DNA (ssDNA), a characteristic marker of cell apoptosis, was tested via the ApoStrandTM ELISA apoptosis detection ELISA kit (BIOMOL International, Plymouth Meeting, PA). The ssDNA ELIAS OD at 450 nm was recorded.

### TUNEL assay

Cells were seeded at 2.5 × 10^4^ cells/cm^2^. Following treatment, cells were stained with TUNEL (10 μM) for 20 min. Cells with positive nuclear TUNEL staining were labeled as apoptotic cells. TUNEL ratio (TUNEL/DAPI× 100%) was recorded from a total of 500 cells from ten random views (1 × 100, Zeiss) for each treatment.

### Annexin V assay

Cells with the applied treatment were harvested, washed, and incubated with Annexin V and propidium iodide (PI) dyes (10 μg/mL, Beyotime Biotechnology, Wuxi, China). Afterwards, cells were analyzed by fluorescent-activated cell sorting (FACS) on the FACSCalibur machine (BD Biosciences). Annexin V ratio was recorded.

### Nrf2 shRNA

Two different lentivirus-packed Nrf2 shRNAs, targeting non-overlapping sequence of *human Nrf2* (sc-37030-V/“shNrf2–1” and sc-44332-V/“shNrf2–2”)*,* as well as the lentiviral *murine Nrf2* shRNA [sc-37049-V, “shNrf2 (m)”] and the scramble nonsense control shRNA (“shC”, sc-108080) were purchased from Santa Cruz Biotech (Santa Cruz, CA). shRNA lentivirus were added to cultured cells in the presence of polybrene (5 μg/mL) for 48 h. Puromycin (1.0 μg/mL) was then included to select stable cells for 4–5 passages. Nrf2 knockdown in the stable cells was confirmed by Western blotting assay and qPCR assay.

### Nrf2 knockout

The lentiCRISPR-GFP-Nrf2-puro KO construct, a gift from Dr. Li [24], was introduced to SH-SY5Y cells via transfection. FACS assay was then performed to sort the GFP-positive cells. Single cells were cultured onto 96-well plate to generate the monoclonal cells. Stable cells were further selected by puromycin. Nrf2 knockout was confirmed by Western blotting assay.

### Keap1 knockout

The Keap1 CRISPR/Cas9 KO Plasmid was purchased from Santa Cruz Biotech (sc-400190-KO-2). The construct was transfected to HEK-293 cells with the lentivirus packaging plasmids, psPAX2 and pMD2.G (provided by Genechem, Shanghai, China) using Lipofectamine 2000 reagent. The lentivirus was harvested at day-3, added to SH-SY5Y cells in the presence of polybrene. Puromycin (1.0 μg/mL) was then included to select stable cells. Keap1 knockout in the stable cells was confirmed by Western blotting assay.

### Keap1 mutation

The in vitro site-directed mutagenesis system (Genechem, Shanghai, China) was applied to generate Cys151S mutant Keap1 vector [25] (GFP-tagged). The construct was sub-cloned into the GV248 lentiviral vector, added to SH-SY5Y cells. Stable cells were selected by puromycin. Expression of the Cys151S Keap1 in stable cells was verified by Western blotting assay.

### Statistical analysis

For each experiment, *n* = 5 (five replicated wells/dishes). Experiments were repeated three to four times. Data of all repeated experiments were pulled together to calculate mean ± standard deviation (SD). Data were analyzed by one-way ANOVA followed by a Scheffe’s f-test via SPSS 18.0 software (SPSS Inc., Chicago, IL). Two-tailed unpaired T test (Excel 2017) was applied to test significance between two treatment groups. Significance was chosen as *P* < 0.05.

## Results

### Four-octyl itaconate activates Nrf2 signaling in neuronal cells

**Fig. 1:**
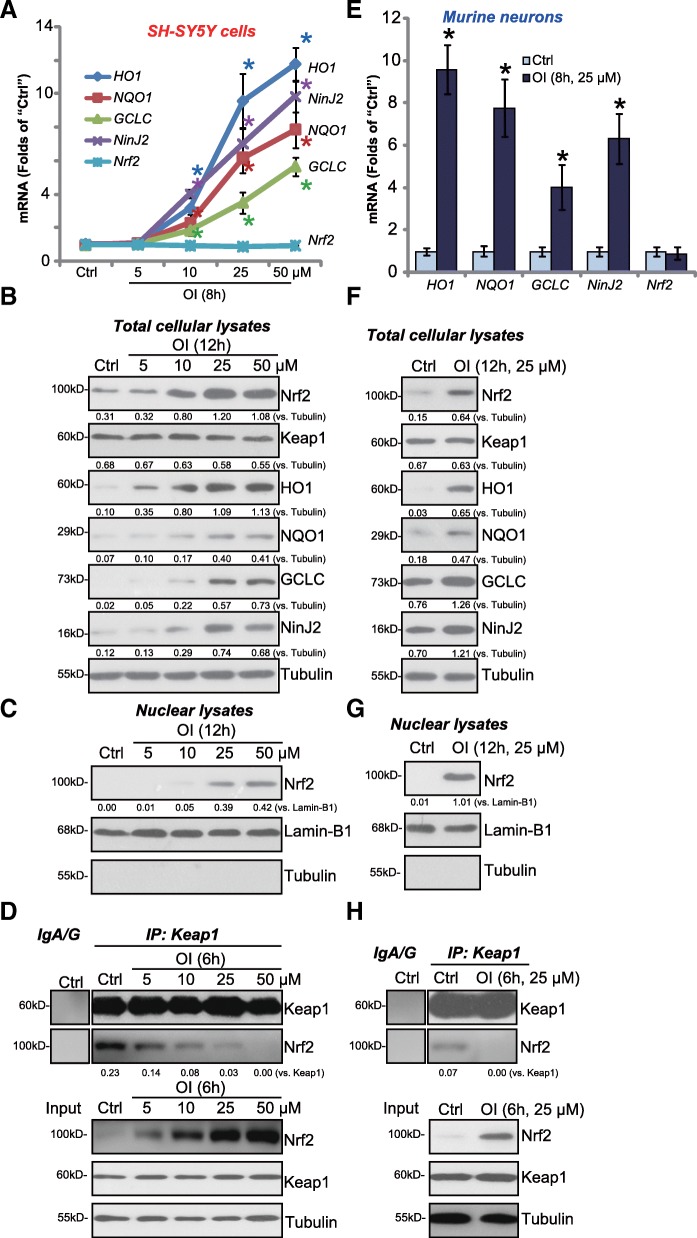
Four-octyl itaconate activates Nrf2 signaling in neuronal cells. SH-SY5Y cells (**a**-**d**) or the primary murine neurons (**e**-**h**) were treated with applied concentration of 4-octyl itaconate (OI) for indicated time, mRNA expression of Nrf2-regulated genes and *Ninj2* were tested by qPCR assay (**a** and **e**); Expression of listed proteins in total cellular lysates (**b** and **f**) and nuclear lysates (**c** and **g**) were tested by the Western blotting assays. Keap1-Nrf2 association was detected by co-immunoprecipitation assays (**d** and **h**). Expression of listed proteins were quantified and normalized to the loading control (**b**, **c**, **f** and **g**). Keap1-bound Nrf2 was quantified as well (**d** and **h**). Lamin-B1 was tested as a marker of nuclear protein (**c** and **g**). “Ctrl” stands for untreated control cells (Same for all Figures). Bars stand for mean ± standard deviation (S.D). * *P* < 0.05 vs. “Ctrl” cells

The effect of 4-octyl itaconate (OI) on Nrf2 signaling was examined. SH-SY5Y human neuronal cells were treated with OI (5–50 μM). By performing the qPCR assay, we show that OI dose-dependently increased mRNA levels of the known Nrf2-dependent genes, including *HO1*, *NQO1* and *GCLC* (Fig. 1a). Western blotting assay results confirm that HO1, NQO1 and GCLC protein levels were elevated as well (Fig. 1b). Although *Nrf2 mRNA* was unchanged (Fig. 1a), the Nrf2 protein level was significantly increased in OI (10–50 μM)-treated SH-SY5Y cells (Fig. 1b). Importantly, stabilized Nrf2 protein translocated to cell nuclei following OI treatment (Fig. 1c), which is a key step for Nrf2 activation [9]. Further co-immunoprecipitation (“IP”) assay results show that Keap1 immunoprecipitated with Nrf2 only in the untreated control SH-SY5Y cells (Fig. 1d). Treatment with OI dose-dependently disrupted Keap1-Nrf2 association (Fig. 1d, “**IP”**), leading to Nrf2 protein stabilization (Fig. 1d, “**Input**”).

Similar experiments were performed in the primary murine neurons. The basal and inducible Nrf2 activities in neurons are extremely low due to epigenetic repression of Nrf2 gene promoter [26]. Therefore, the primary neurons were cultured in TSA (a histone deacetylase inhibitor)-containing medium for 10 days (DIV10). As shown, OI (25 μM) induced Nrf2-dependent gene (*HO1*, *NQO1* and *GCLC*) expression (Fig. 1e and f), Nrf2 protein stabilization (Fig. 1f) and nuclear translocation (Fig. 1g). Keap1-Nrf2 association was disrupted by OI as well, as Keap1-bound Nrf2 was largely decreased (Fig. 1h). These results indicate that OI activates Nrf2 signaling in the neuronal cells. Notably, Keap1 expression was unchanged by OI (Fig. 1b, d, f and h). A time-dependent response by OI was tested. Results showed that significant Nrf2 protein stabilization as well as HO1, and GCLC protein expression were detected as early as 1 h following OI treatment in primary neurons (Additional file 1: Figure S1). Importantly, without TSA presence, basal and inducible (by OI) Nrf2 activities in DIV10 neurons were negligible (Additional file 1: Figure S1). Keap1 levels were indifferent (Additional file 1: Figure S1).

Ninjurin2 (Ninj2), a homolog of ninjurin1 (Ninj1), is a homophilic cellular adhesion molecule [27]. Ninj2 is expressed in neurons to promote neurite outgrowth [27]. Our results show that after OI (10–50 μM) treatment *Ninj2 mRNA* and protein levels were significantly elevated in SH-SY5Y cells (Fig. 1a and b) and in primary neurons (Fig. 1e and f). These results imply that Ninj2 can be induced following Nrf2 activation by OI in neuronal cells.

### Four-octyl itaconate attenuates H_2_O_2_-induced neuronal cell death and apoptosis

**Fig. 2:**
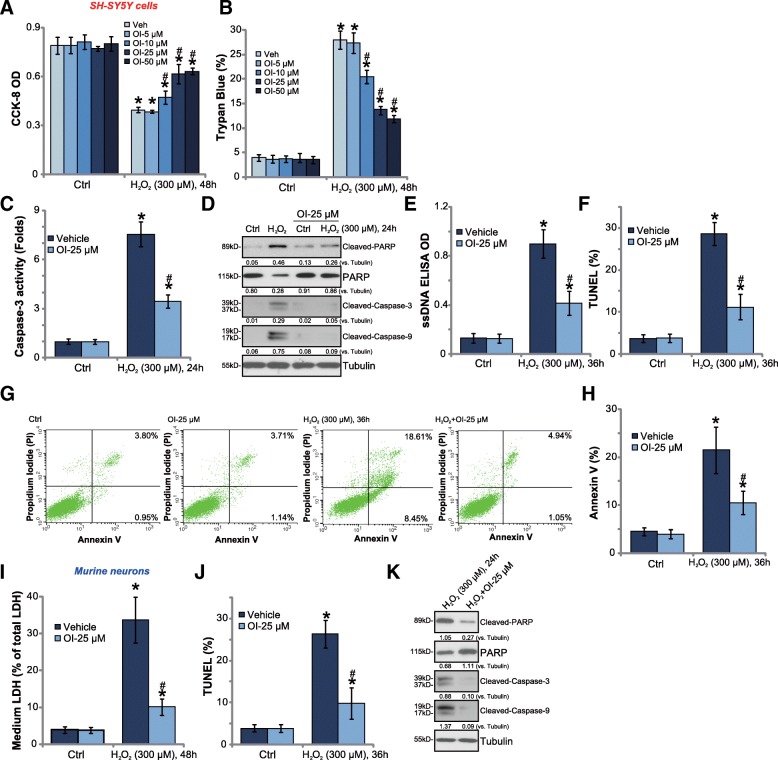
Four-octyl itaconate attenuates H_2_O_2_-induced neuronal cell death and apoptosis. SH-SY5Y cells (**a**-**h**) or the primary murine neurons (**i**-**k**) were pretreated for 30 min with applied concentration of 4-octyl itaconate (OI), followed by stimulation of H_2_O_2_ (300 μM) for indicated time, cell viability, cell death and apoptosis were tested by the listed assays mentioned in the text (**a**-**k**). Expression of listed proteins were quantified and normalized to the loading control (**d** and **k**). Bars stand for mean ± standard deviation (S.D., *n* = 5). * *P* < 0.05 vs. “Ctrl” cells. ^#^ *P* < 0.05 vs. H_2_O_2_ treatment only

Nrf2 activation can protect neuronal cells from oxidative stress [28–31]. H_2_O_2_ (300 μM) treatment in SH-SY5Y neuronal cells induced significant cell viability (CCK-8 OD) reduction (Fig. 2a) and cell death (Trypan blue increase, Fig. 2b). Significantly, pretreatment with OI dose-dependently inhibited H_2_O_2_-induced cytotoxicity in SH-SY5Y cells (Fig. 2a-b). OI single treatment was non-cytotoxic (Fig. 2a and b). Among the tested concentrations, OI at 25 μM efficiently protected SH-SY5Y cells from H_2_O_2_ (Fig. 2a and b). This concentration was chosen for further experiments.

The potential effect of OI on cell apoptosis was tested next. In SH-SY5Y cells, H_2_O_2_ (300 μM) treatment induced caspase-3 activation (Fig. 2c), PARP [poly (ADP-ribose) polymerase], caspase-3 and caspase-9 cleavages (Fig. 2d), as well as single strand DNA (ssDNA) accumulation (Fig. 2e) and TUNEL-staining ratio increase (Fig. 2f), which were significantly attenuated by OI pretreatment (Fig. 2c-f). By performing the Annexin V-PI FACS assay, we further show that H_2_O_2_-induced SH-SY5Y cell apoptosis, or the Annexin V ratio increase, was significantly inhibited by OI (Fig. 2g and h). These results convincingly show that OI efficiently inhibits H_2_O_2_-induced SH-SY5Y cell apoptosis.

In the primary murine neurons, pretreatment with OI (25 μM) alleviated H_2_O_2_-induced cell death (LDH medium release, Fig. 2i) and apoptosis (TUNEL ratio increase, Fig. 2j). Additionally, H_2_O_2_-induced cleavages of PARP, caspase-3 and caspase-9 were attenuated as well by OI (25 μM) (Fig. 2k). These results further confirmed the neuroprotective function of OI against H_2_O_2_. OI alone was ineffective in the neuronal cells (Fig. 2a-j).

### OI inhibits H_2_O_2_-induced oxidative stress in neuronal cells

**Fig. 3:**
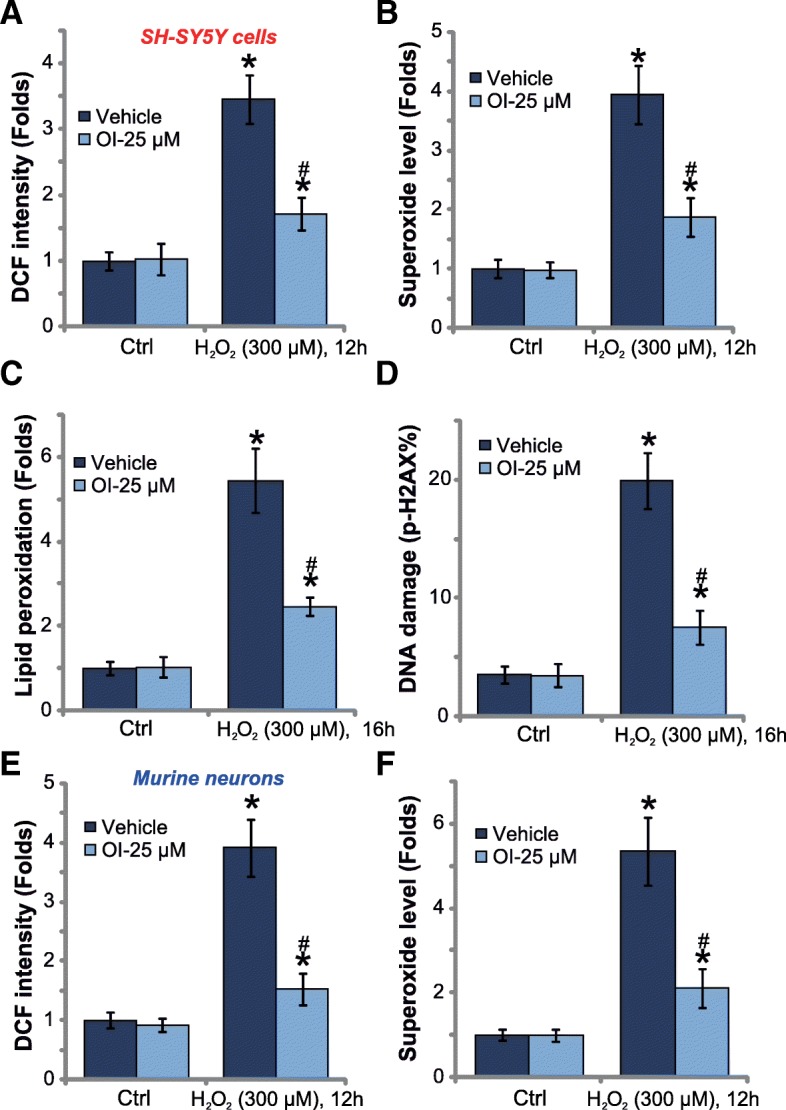
OI inhibits H_2_O_2_-induced oxidative stress in neuronal cells. SH-SY5Y cells (**a**-**d**) or the primary murine neurons (**e**-**f**) were pretreated for 30 min with 4-octyl itaconate (OI, 25 μM), followed by stimulation of H_2_O_2_ (300 μM) for indicated time, relative DCF fluorescent intensity (**a** and **e**) and relative superoxide level (**b** and **f**) were tested; The lipid peroxidation (**c**) and DNA damage (**d**) were tested by TBAR activity assay and p-H2AX FACS assay, respectively. Bars stand for mean ± standard deviation (S.D., n = 5). “Vehicle” stands for PBS control (Same for all Figures). * *P* < 0.05 vs. “Ctrl” cells. ^#^ *P* < 0.05 vs. H_2_O_2_ treatment only

Increases of DCF fluorescent intensity imply ROS production [17]. By performing the carboxy-H2DCF-DA dye assay, we suggest that OI pretreatment largely attenuated H_2_O_2_-induced ROS production in SH-SY5Y cells (Fig. 3a). Furthermore, H_2_O_2_-induced superoxide accumulation (Fig. 3b) was inhibited by OI as well. Consequently, H_2_O_2_-induced lipid peroxidation (TBAR activity increase [17], Fig. 3c) and DNA damages (p-H2AX ratio increase, Fig. 3d) were significantly alleviated. These results suggest that OI inhibited H_2_O_2_-induced oxidative stress in SH-SY5Y cells. In the primary murine neurons, pretreatment with OI (25 μM) similarly inhibited H_2_O_2_-induced ROS production (DCF fluorescent intensity, Fig. 3e), superoxide accumulation (Fig. 3f). Thus, OI, the novel Nrf2 activator, potently inhibits H_2_O_2_-induced oxidative stress in neuronal cells.

### Nrf2 activation mediates 4-octyl itaconate-induced neuronal cell protection against H_2_O_2_

**Fig. 4:**
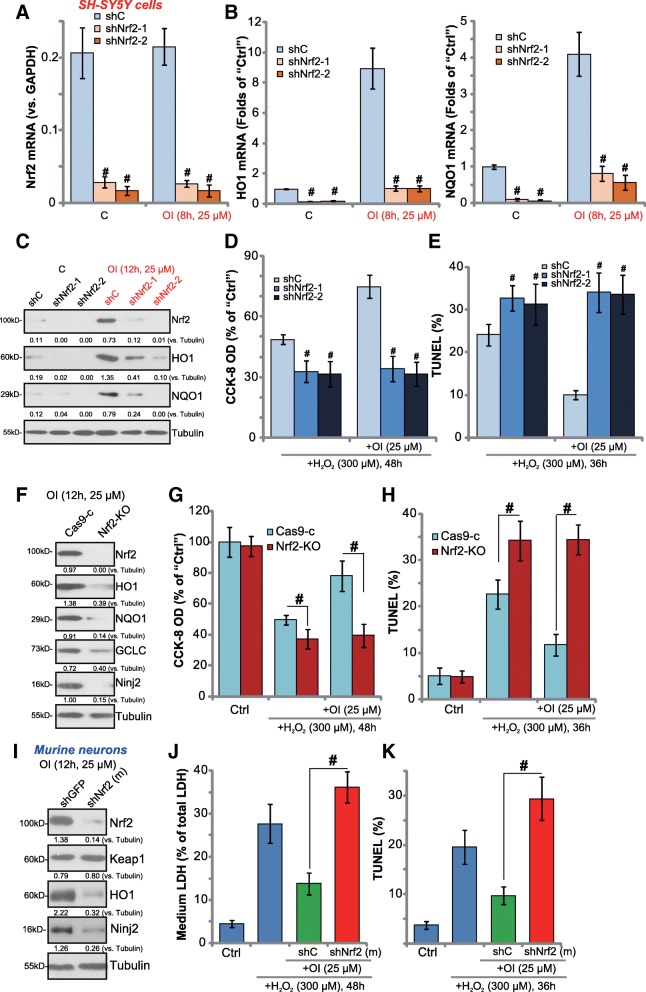
Nrf2 activation mediates 4-octyl itaconate-induced neuronal cell protection against H_2_O_2_. SH-SY5Y cells (**a**-**e**) or the primary murine neurons (**i**-**k**), with the applied Nrf2 shRNA or the scramble control shRNA (“shC”), were either untreated or treated with 4-octyl itaconate (OI), mRNA and protein expression of listed genes were shown (**a**-**c**, and **i**); Cells were pretreated for 30 min with OI (25 μM), followed by stimulation of H_2_O_2_ (300 μM) for indicated time, cell viability (CCK-8 OD, **d**), cell death (LDH release, **j**) and apoptosis (TUNEL ratio increase, **e**, and **k**) were tested. Stable SH-SY5Y cells, with the CRISPR/Cas9-Nrf2 KO construct (“Nrf2-KO”) or the CRISPR/Cas9 control construct (“Cas9-c”), were treated with 4-octyl itaconate (OI), listed proteins were shown (**f**); Cells were pretreated for 30 min with OI (25 μM), followed by stimulation of H_2_O_2_ (300 μM) for indicated time, cell viability (**g**) and apoptosis (**h**) were tested. Expression of listed proteins were quantified and normalized to the loading control (**c**, **f** and **i**). “shNrf2 (m)” stands for murine Nrf2 shRNA (**I**-**K**). Bars stand for mean ± standard deviation (S.D., n = 5). ^#^ *P* < 0.05 vs. “shC” cells (**a**, **b**, **d** and **e**). ^#^ *P* < 0.05 (**g**, **h**, **j** and **k**)

To study the link between Nrf2 activation and OI-induced neuroprotection, shRNA strategy was employed to silence Nrf2. As described, the lentivirus encoding Nrf2 shRNA (“shNrf2–1” or “shNrf2–2”, with non-overlapping sequence) were added to proliferating SH-SY5Y cells. After selection by puromycin, stable cells with Nrf2 shRNA were established. Results show that *Nrf2 mRNA* and protein levels were significantly downregulated in Nrf2 shRNA-expressing stable cells, with/without OI treatment (Fig. 4a). Basal and OI-induced mRNA expression of *HO1* and *NQO1* were largely inhibited the Nrf2 shRNA (Fig. 4b). OI-induced Nrf2 protein stabilization as well as HO1 and NQO1 protein expression were also significantly attenuated by the Nrf2 shRNA (Fig. 4c). GCLC and Ninj2 mRNA and protein expression in response to OI were blocked as well by the Nrf2 shRNA in SH-SY5Y cells (Data not shown).

Importantly, in Nrf2-silenced SH-SY5Y cells, OI was unable to inhibit H_2_O_2_-induced viability reduction (Fig. 4d) and cell apoptosis (Fig. 4e). Therefore, OI was ineffective against H_2_O_2_ when Nrf2 was silenced (Fig. 4d and e). Notably, the Nrf2-silenced SH-SY5Y cells were more vulnerable to H_2_O_2_, showing intensified cell death and apoptosis (as compared to control cells, Fig. 4d and e). Nrf2 shRNA alone did not affect SH-SY5Y cell death/apoptosis (Data not shown).

Next, CRISPR/Cas9 method was employed to completely knockout Nrf2 in SH-SY5Y cells, and stable cells were established. As shown, Nrf2 protein was completely depleted in cells with the CRISPR/Cas9-Nrf2 KO construct (“Nrf2-KO” cells) (Fig. 4f). OI-induced HO1, NQO1, GCLC and Ninj2 protein expression were nullified in Nrf2-KO cells (Fig. 4f). Similar to the shRNA results, H_2_O_2_-induced cell death (viability reduction, Fig. 4g) and apoptosis (Fig. 4h) were largely potentiated in Nrf2-KO cells (vs. the CRISPR/Cas9-control cells, “Cas9-c”). Significantly, adding OI was unable to protect Nrf2-KO cells from H_2_O_2_ (Fig. 4g and h). It was only effective in the control “Cas9-c” cells. Thus, Nrf2 KO abolished OI-induced actions in SH-SY5Y cells (Fig. 4g and h). These results further suggest that Nrf2 activation mediates OI-induced SH-SY5Y cell protection against H_2_O_2_.

In the primary murine neurons, adding Nrf2 shRNA [“shNrf2 (m)”]-containing lentivirus efficiently downregulated Nrf2 (Fig. 4i), which inhibited OI-induced expression of HO1 and Ninj2 (Fig. 4i). Significantly, Nrf2 shRNA also abolished OI-induced neuronal cell protection against H_2_O_2_ (Fig. 4j and k). These results again support that activation of Nrf2 mediates OI-mediated neuroprotection.

### Keap1 knockout or Cys151S mutation abolishes 4-octyl itaconate-induced neuroprotection against H_2_O_2_

**Fig. 5:**
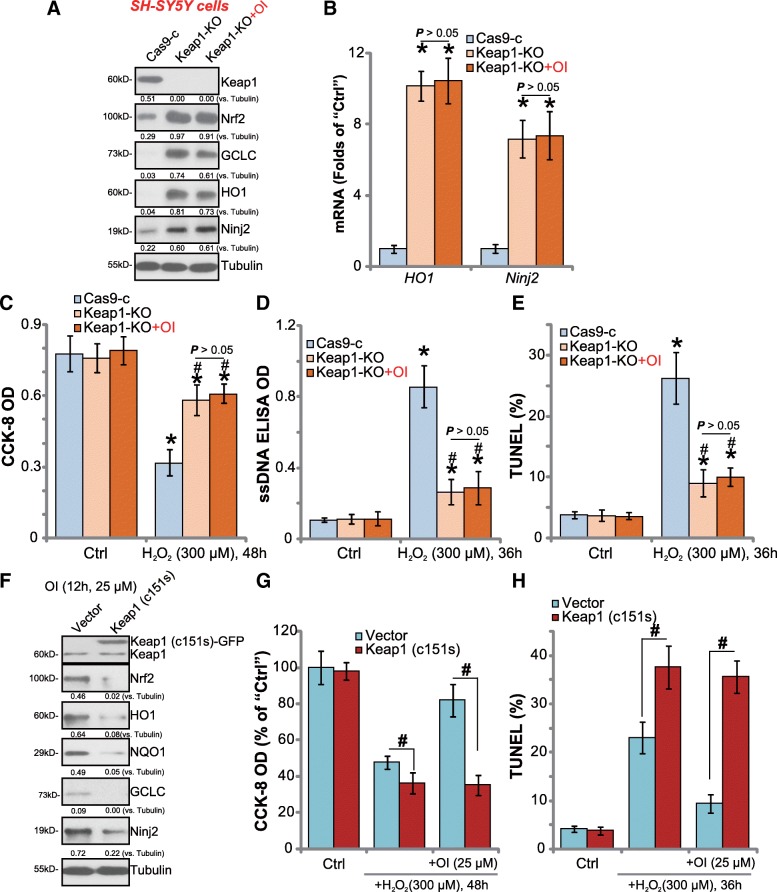
Keap1 knockout or Cys151S mutation abolishes 4-octyl itaconate-induced neuroprotection against H_2_O_2_. Stable SH-SY5Y cells with the lentiviral CRISPR/Cas9 Keap1 vector (“Keap1-KO”) or the CRISPR/Cas9 control vector (“Cas9-c”) were treated with/without 4-octyl itaconate (“+OI”, 25 μM, for 12 h), mRNA and protein expression of listed genes were shown (**a** and **b**). Cells were pretreated with/without OI (“+OI”, 25 μM), followed by stimulation of H_2_O_2_ (300 μM) for indicated time, cell viability (**c**) and apoptosis (**d** and **e**) were tested by the listed assays. Stable SH-SY5Y cells with the Cys151S mutant Keap1 [“Keap1 (c151s)”] or the empty vector (“Vector”) were treated with 4-octyl itaconate (“OI”, 25 μM) for 12 h, expression of listed proteins were shown (**f**). Cells were pretreated with/without OI (“+OI”, 25 μM), followed by stimulation of H_2_O_2_ (300 μM) for indicated time, cell viability (**g**), and apoptosis (**h**) were tested. Expression of listed proteins were quantified and normalized to the loading control (**a** and **f**). Bars stand for mean ± standard deviation (S.D., n = 5). * *P* < 0.05 vs. “Cas9-c” cells (**b**). * *P* < 0.05 vs. “Ctrl” cells (**c**-**e**). ^#^ *P* < 0.05 vs. H_2_O_2_ treatment of “Cas9-c” cells (**c**-**e**). ^#^ *P* < 0.05 (**g** and **h**)

It has been shown that OI alkylates Keap1, causing Keap1-Nrf2 disassociation, Nrf2 stabilization and activation [14]. If Keap1 is the primary target of OI, Keap1 depletion should abolish OI-induced actions in neuronal cells. To test this hypothesis, CRISPR/Cas9 gene editing method was again employed. As described, the lentiviral CRISPR/Cas9 Keap1 KO vector was transfected to SH-SY5Y cells. Via selection, stable cells with the construct were established (“Keap1-KO” cells). By performing the Western blotting assay, we confirmed that Keap1 protein was completely depleted in the stable cells (Fig. 5a), where Nrf2 protein level was significantly elevated (Fig. 5a). HO1, GCLC and Ninj2 protein expression were significantly increased as well in Keap1-KO cells (Fig. 5a), where *HO1* and *Ninj2* mRNA levels were significantly higher (Fig. 5b).

As expected, Keap1 KO largely inhibited H_2_O_2_ (300 μM)-induced SH-SY5Y cell viability reduction (Fig. 5c) and apoptosis (Fig. 5d and e). Significantly, adding OI in the Keap1-KO cells failed to further boost Nrf2 signaling (Fig. 5a and b). More importantly, OI was unable to further protect Keap1-KO cells from H_2_O_2_ (Fig. 5c-e). Therefore, Keap1 depletion mimicked and abolished OI-induced actions in neuronal cells.

The above results suggest that Keap1 should be the primary target of OI. Itaconate directly alkylates Keap1 at cysteine-151 (Cys151) and other cysteine residues, essential for Nrf2 departure and activation. Thus, a Cys151S mutant Keap1 [14] vector was transfected to SH-SY5Y cells. Stable cells were established again via puromycin selection. Western blotting assay results confirmed the expression of mutant Keap1 (GFP-tagged) in the stable cells [“Keap1 (c151s)”, Fig. 5f]. Significantly, OI-induced Nrf2 stabilization as well as HO1, NQO1, GCLC and Ninj2 protein expression were almost blocked in Keap1-mutant cells (Fig. 5f). Importantly, OI-induced SH-SY5Y cytoprotection against H_2_O_2_ was significantly inhibited in cells with Cys151S-mutant Keap1 (Fig. 5g and h). As compared to the vector control cells, Keap1-mutant cells were more sensitive to H_2_O_2_-induced damage (Fig. 5g and h). These results indicate that Keap1 alkylatation and following Nrf2 activation could be the primary mechanism of OI-induced neuroprotection against H_2_O_2_.

## Discussion

Oxidative stress-induced neuronal cell injury contributes significantly to the pathogenesis of neurodegenerative diseases [4, 32, 33]. Among various therapeutic strategies, one promising method is to boost the endogenous defense mechanisms (i.e. Nrf2 signaling) against oxidative stress through pharmacological intake of small compounds [11–13]. Activated Nrf2 separates separates from Keap1, enters to cell nuclei, and binds to ARE to promote transcription and expression of multiple antioxidant enzymes and detoxifying genes, thereby inhibiting neuronal oxidative injury [11–13]. Many Nrf2 activators have been proven to be strong radical scavengers, but often have some severe adverse effects and poor bioavailability [34, 35]. Recently, the research attention has been focusing on searching for novel Nrf2 activators that can scavenge free radicals and efficiently protect neuronal cells from oxidative injury [34, 35].

In the current study, we show that OI, the cell-permeable derivative of itaconate [14], activated Nrf2 signaling in SH-SY5Y cells and primary murine neurons. OI induced Keap1-Nrf2 disassociation, Nrf2 protein stabilization and nuclear translocation, leading to expression of multiple known Nrf2 target genes. Importantly, OI pretreatment potently attenuated H_2_O_2_-induced ROS production, oxidative stress, lipid peroxidation and DNA damage. As a result, H_2_O_2_-induced neuronal cell death and apoptosis were significantly attenuated. These results show that activation of Nrf2 by OI should be a fine strategy to protect neurons/neuronal cells from oxidative stress.

Ninjurin2 (Ninj2) is a homolog of Ninj1 [27]. It is a adhesion molecule expressed in neurons [27]. Ninj2 functions in neurons are not fully understood. In the current study, we show that basal Ninj2 expression is low in SH-SY5Y cells and murine neurons. OI significantly elevated *Ninj2 mRNA* and protein expression. Importantly, OI-induced Ninj2 expression was almost blocked by Nrf2-shRNA/−knockout or Keap1 mutation. Furthermore, Keap1 knockout induced Ninj2 expression in SH-SY5Y cells. These results suggest that *Ninj2* could possibly be a novel Nrf2-regulated gene that can be induced by OI in neuronal cells. Our results provide novel molecular insights to possibly explain the established link between *Ninj2* polymorphism and ischemic stroke [36–38]. It will be interesting to further explore the underlying mechanism of OI-induced Ninj2 expression, as well as the possible anti-oxidant and neuroprotective functions of Ninj2.

Nrf2-Keap1 is the primary target of OI. Nrf2 knockdown by targeted-shRNA or CRISPR/Cas9 Nrf2 KO almost abolished OI-induced neuronal cell protection against H_2_O_2_. Further, OI was ineffective in Keap1-KO cells where Nrf2 is over-activated. OI alkylates Keap1 to block Keap1-Nrf2 association [14]. This shall lead to robust and sustained Nrf2 activation. Indeed, we show that ectopic overexpression of a Cys151S mutant Keap1 in SH-SY5Y cells reversed OI-induced Nrf2 activation and anti-H_2_O_2_ neuroprotection. These genetic evidence suggest that Keap1-Nrf2 should be the primary target of OI in neuronal cells.

## Conclusions

OI activates Keap1-Nrf2 signaling to protect SH-SY5Y cells and epigenetically de-repressed primary neurons from H_2_O_2_ in vitro. Bell et al.*,* reported that basal and inducible Nrf2 activities in mature neurons are extremely low following epigenetic repression of Nrf2 gene’s promoter in the process of development [26]. Hence, in this study epigenetic de-repression (by TSA) is required to observe Nrf2 induction in neurons in culture. Without TSA, basal and inducible (by OI) Nrf2 activities were negligible. Therefore, results obtained in this study are only applicable to the in vitro conditions, and might have limited relevance for the in vivo situation, where Nrf2 should still be repressed in neurons. It would be interesting to test the potential effect of OI in vivo, especially considering that Nrf2 activation in cultured neurons is dependent on the presence of astrocytes, where Nrf2 is intact [26].

## Additional file

Additional file 1: **Figure S1.** The primary murine neurons, cultured in medium with/without TSA (20 ng/mL) for 10 days (DIV10), were treated with 4-octyl itaconate (OI, 25 μM) for indicated time periods, expression of listed proteins in total cellular lysates were tested by the Western blotting assays. (EPS 1034 kb)

## Abbreviations

CCK-8: Cell Counting Kit-8
CNS: Central nerve system
FBS: Fetal bovine serum
GCLC: γ-glutamyl cysteine ligase catalytic subunit
H_2_O_2_: Hydrogen peroxide
HO-1: Heme oxygenase 1
Keap1: Kelch like ECH-associated protein 1
LDH: Lactate dehydrogenase
Ninj2: Ninjurin2
NQO1: NAD(P) H quinone oxidoreductase-1
Nrf2: Nuclear factor E2-related factor 2
OD: Optical density
OI: Four-octyl itaconate
PI: Propidium iodide
qPCR: Quantitative real-time PCR
ROS: Reactive oxygen species
SD: Standard deviation
TBAR: Thiobarbituric acid reactive substances
TUNEL: TdT-mediated dUTP nick end labeling
